# Highly Durable Antibacterial Properties of Cellulosic Fabric via β-Cyclodextrin/Essential Oils Inclusion Complex

**DOI:** 10.3390/polym14224899

**Published:** 2022-11-13

**Authors:** A. Farouk, S. Sharaf, R. Refaie, M. M. Abd El-Hady

**Affiliations:** 1 National Research Centre, Textile Research and Technology Institute, 33 El-Behoth Street, Dokki, Cairo P.O. Box 12622, Egypt; 2 Department of Chemistry, Faculty of Science, King Khalid University, Abha 62529, Saudi Arabia; 3 Department of Physics, College of Science and Arts in Al-Asyah, Qassim University, Buraidah 51452, Saudi Arabia

**Keywords:** cotton fabric, essential oil, β-cyclodextrin, antibacterial activity, durability, air permeability

## Abstract

Essential oils (EO_S_), which naturally come from plants, have significant antibacterial properties against a variety of pathogens, but their high volatility and poor water solubility severely restrict their use in the textile industry. In this study, an inclusion complex based on β-cyclodextrin (β-CD)/EO_S_ was prepared by two different simple methods: pad dry cure (method 1) and pad batch (method 2). A glyoxal crosslinking agent was used for the fixation of the inclusion complexes on the surface of the fabric. Lavender, rosemary, salvia, and lemon essential oils were applied. The structure of the β-CD/EOs inclusion complex was investigated using scanning electron microscopy (SEM), Fourier transform infrared spectroscopy (FTIR), and weight gain (%), which indicated that the β-CD/EOs were successfully deposited on cellulose-based fabric. The results demonstrated that β-CD enhanced the oils’ scent stability, with the advantage of exhibiting no major change in the tensile strength or permeability of cotton. Lavender oil had the highest stability scent with a value of 3.25, even after 30 days of evaluation. The antibacterial activity showed that EO_S_/βCD-impregnated fabrics for method 1 had an inhibition zone ranging from 33 to 23 mm, while the inhibition zone for method 2 ranged from 39 mm to 29 mm, indicating that our treatment was able to control the growth of bacteria, even after five washing cycles. This study confirmed that the EOs/βCD inclusion-complex-deposited cotton fabric might hold further promise for medical and hospital use.

## 1. Introduction

Oils extracted from aromatic plants are called essential oils, and they contain volatile compounds with various biological effects. They have an essential role in the textile industry and are a key representative of natural antibacterial agents. Additionally, they are used in the manufacturing of high-value-added products such as cosmetic textiles; and medical textiles that are biocompatible, ecologically friendly, and nontoxic, and that feature antibacterial, anticancer, antioxidant, and aroma therapeutic characteristics [1,2,3,4]. However, due to their instability, ease of decomposition, and volatilization [5], EO_S_ have only a limited extent of potential use in textiles. These oils are complicated mixtures of more than 300 different compounds [6]. They are partially in the vapor state because their vapor pressure is high enough at atmospheric pressure and room temperature [7]. These volatile compounds correspond to a variety of chemical groups, including alcohols, ethers, aldehydes, ketones, phenols, heterocycles, and, in particular, terpenes. Alcohols, aldehydes, and ketones offer an extensive array of aromatic compounds [8]. 

The variation in chemical compounds and their percentage in the essential oil from natural sources depend on geographical distribution, as well as environmental conditions such as temperature [9]. Lavender essential oil contains several antimicrobial compounds. The main active ingredients are monoterpenes (linalool, linalyl acetate, lavandulol, geraniol, bornyl acetate, borneol, terpineol, and eucalyptol or lavandulyl acetate). High and almost equal contents of linalool and linalyl acetate (a ratio above one) are required for good antimicrobial properties of lavender essential oil [10]. The polyphenolic profile of rosemary is characterized by the presence of carnosic acid, carnosol, rosmarinic acid, and hesperidin, as major components [11]. The main compounds of Salvia officinalis essential oil were reported as follows: α-thujone, (E)-β-caryophyllene, 1,8-cineole, α-humulene, β-pinene, thujone, camphor, allo-aromadendrene, borneol, and α-pinene [12]. Twenty-one components were identified in lemon oil, and the dominant compounds were citral, limonene and β-pinene [13].

Microencapsulation technology can overcome this problem by preserving essential oils from the external environment, improving their stability and providing a stable platform for the further development and use of essential oils [14]. It describes the process of encasing the target substance, which may be a gas, a liquid droplet, or a solid particle, in small containers with core–shell structures that range in size from several hundred to thousands of microns in diameter [15]. Cyclodextrins (CDs) are the cyclic oligosaccharides made when bacteria such as Bacillus macerans break down starch. The most notable characteristic of the CDs is their capacity to molecularly bind with chemicals to create solid inclusion complexes (host–guest complexes). The complex between the host and the guest is created when the guest molecule fits into the host’s cavity. During the formation of the inclusion complex, no covalent bonds are broken or made, and hydrophobic interactions may be the main force behind CD-based host–guest complexes [16]. As is clear from the information provided above, the cyclodextrin molecule can be incorporated into other active compounds, such as fragrances, medications, fungicides, or bactericidal agents, in a way that results in inclusion complexes. Complexing agents are slowly released from cyclodextrin cavities [17,18,19]. Due to their chemical composition and ability to form inclusion complexes with molecules of various active substances, cyclodextrins play an essential role in the field of medical textiles. Because nontoxicity and antimicrobial efficacy are necessary, many chemicals are incorporated into cyclodextrin cavities and then coated to the fabric surface. Cyclodextrins are frequently employed to develop a complex with EOs that have therapeutic effects and low environmental impact. Spraying, printing, padding, grafting, surface coating, impregnation, inkjet printing, and sol-gel can be used to insert β-CD into textiles [20]. The potential for permanent crosslinking between inclusion complexes of β-cyclodextrins and cellulose substrate, which develops interactions between the reactive group of β-CD and hydroxyl groups of cellulose, has been previously reported [21,22,23,24,25,26].

To the best of our knowledge, all literature points to the use of modified cyclodextrins for permeant fixation on substrate materials, but little work has been performed on the preparation of crosslinking material for the fixation of cyclodextrin inclusion complexes on the surface of cotton fabric. With this in mind, this work examined the feasibility of combining beta cyclodextrin (β-CD) and essential oils (EOs) to create inclusion complexes aimed at promoting highly durable antibacterial activity. Four different essential oils, namely lavender, rosemary, salvia, and lemon, were applied using two simple methods. The feasibility of binding the prepared complexes for the cellulosic textile, with the aid of glyoxal as the crosslinking agent, was studied. Two different methods were applied for the impregnation of the inclusion complex using pad–dry–cure (method 1) and pad–batch (method 2). The mechanical properties as well as the scent analysis of treated samples were also investigated, which were found to be extremely efficient for the biomedical field.

## 2. Experimental Section

### 2.1. Materials

Bleached plain-woven 100% cotton fabric (138 g/m^2^) was supplied by Misr Company for spinning and weaving (Mehalla El-Kobra, Egypt). 

### 2.2. Chemicals

Lavender, rosemary, salvia, and lemon essential oils were purchased from Sigma Aldrich (St. Louis, MI, USA). β-CD was purchased from Junsei Chemical Ltd, (Tokyo, Japan). Glyoxal, magnesium chloride, and Tween 80 (polyoxyethylene sorbitan monooleate) were supplied by Sigma-Aldrich.

### 2.3. Preparation of Fabrics-Coated β-CD/Eos Inclusion Complex

#### 2.3.1. Method (1) (Pad-Dry–Cure)

**Scheme 1:**
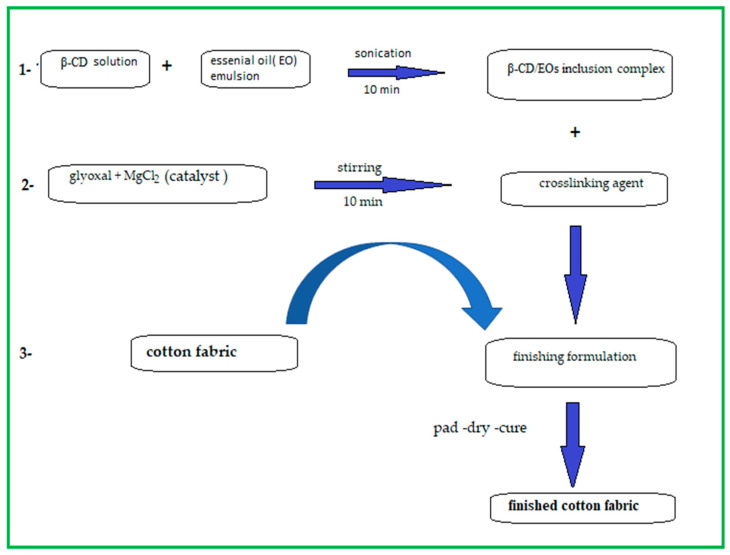
Method 1 (pad-dry- cure).

The preparation of the β-CD/Eos inclusion complex was carried out by mixing 8% β-CD (previously soluble in water at 60 °C for 30 min) and 5 mL of different essential oils (previously stirred with 10 mL Tween 80 emulsifier for 24 h). Then, the mixture was sonicated (Sonics VCX500, 500 W, 20 kHz, Newtown, CT, USA) for 10 min, with a 40% amplitude to obtain the inclusion complex. We prepared, mixed, and stirred 4% crosslinking agent glyoxal and 4% MgCl_2_ catalyst solutions for 10 min, then added to the previous inclusion complex. Finally, samples were immersed in the β-CD/EOs inclusion complex for 10 min, then pad-dried at 80 °C for 5 min and, finally, cured at 130 °C for 3 min (Scheme 1).

#### 2.3.2. Method (2) (Pad-Batch)

**Scheme 2:**
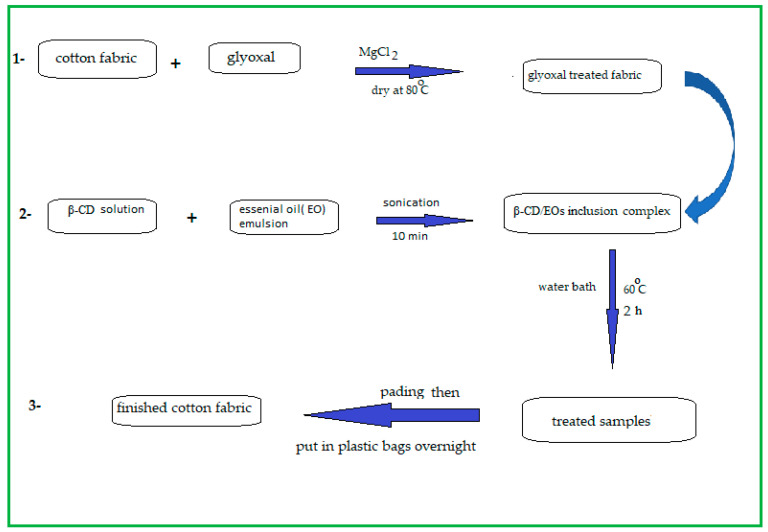
Method (2): pad-batch.

A total of 8% β-CD (previously soluble in water at 60 °C for 30 min) was mixed with 2% of different essential oils (previously stirred with 10 mL of Tween 80 emulsifier for 24 h) to form the β-CD/EOs inclusion complex. Cotton fabrics were immersed for 10 min in 4% crosslinking agent glyoxal and 4% MgCl_2_ catalyst solutions, which were prepared, mixed, and stirred for 10 min. The fabrics were then immersed in the different β-CD/EOs inclusion complexes. The solutions were placed in a water bath at 60 °C for two hours, then fabrics were kept in plastic bags overnight. Finally, a drying process at 60 °C was performed (Scheme 2).

## 3. Characterization

### 3.1. Fourier Transform Infrared Spectroscopy (FTIR)

An FTIR instrument (JASCO, Model IR 4700, Tokyo, Japan) was used, which scanned from 4000 to 400 cm^−1^ in ATR mode using KBr as the supporting material.

### 3.2. Scanning Electron Microscopy (SEM)/EDX Analysis and Visual Color

Samples for SEM/EDX were taken using an FEI INSPECTS Company, Philips, Eindhoven, Holland through environmental scanning without coating. An elemental analysis of the particles was implemented by an SEM equipped with an energy-dispersive spectroscope (EDX), to rapidly quantitatively and qualitatively analyzed the elemental composition.

### 3.3. Antibacterial Analysis

The antibacterial activity of the treated samples against *Staphylococcus aureus*, *Bacillus subtilis* (G+ve), *Escherichia coli*, and *Pseudomonas aeruginosa* (G−ve) bacteria were determined using an agar plate. The diameter of the inhibition zone was determined according to the AATCC test method 100–199.

### 3.4. Weight Gain

The weight gain (%) loading was calculated as follows:

(1) $$\mathrm{Wight}\;\mathrm{gain}\;\left(\%\right)=\frac{W_{2}-W_{1}}{W_{1}}\times 100$$

where *W*_1_ and *W*_2_ are the weights of the samples before and after treatment, respectively.

### 3.5. Durability Test

The treated samples were examined for five laundering cycles, in accordance with the ASTM standard test method (D 737-109 96), to measure the washing resistance of the antibacterial application

### 3.6. Scent Intensity

Four judges in good health were chosen for this evaluation. Every five days throughout the duration of six weeks, the judges smelled the samples. After each wash, the aroma intensity of the specimen was recorded. The rating scale utilized was an ordinal scale, with 5 representing a very strong aroma and 0 representing a loss of scent.

### 3.7. Air Permeability

An essential characteristic of the textile materials used to determine the breathability of coated fabrics is their air permeability. A model for testing air permeability was used to test it (MO21A). In this test, a circle sample of the fabric was attached to the inside of the tester, and the fabric’s air permeability was assessed by passing high air pressure through it and measuring the rate of air flow.

### 3.8. Tensile Strength

Using the ASTM test method D-1682-94 (1994), the tensile strength of the fabric was measured.

### 3.9. Statistical Analysis

The results are expressed as the mean value and standard deviation (mean SD) of each repeated sample (*n* = 3).

## 4. Results and Discussion

### 4.1. FTIR Analysis

**Figure 1:**
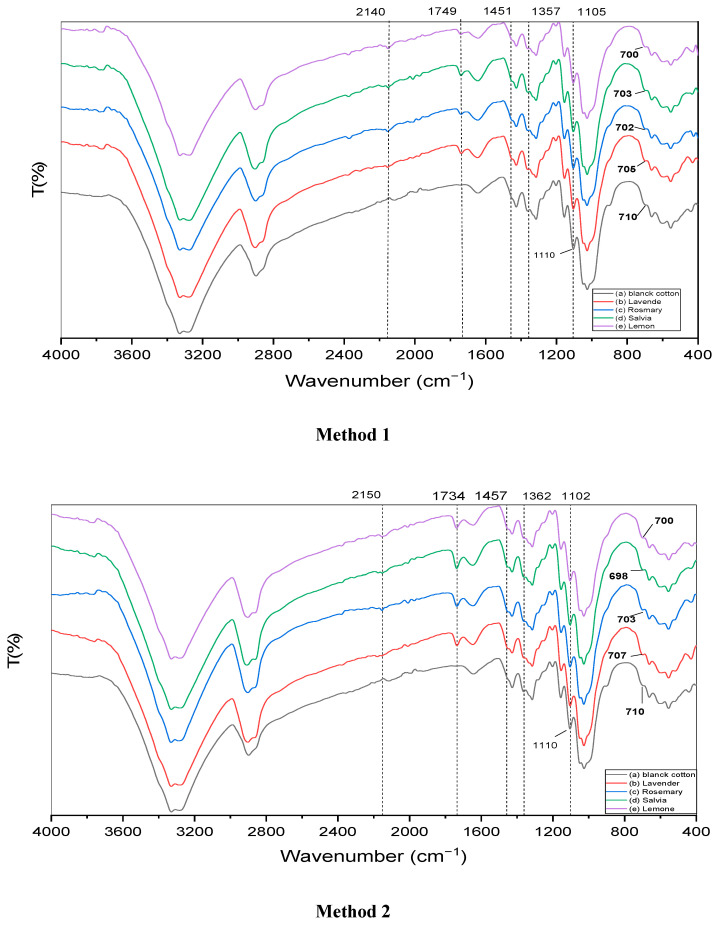
FTIR spectrum of treated cotton fabrics with inclusion complex β-CD-EO_S_ via methods 1 and 2.

**Scheme 3:**
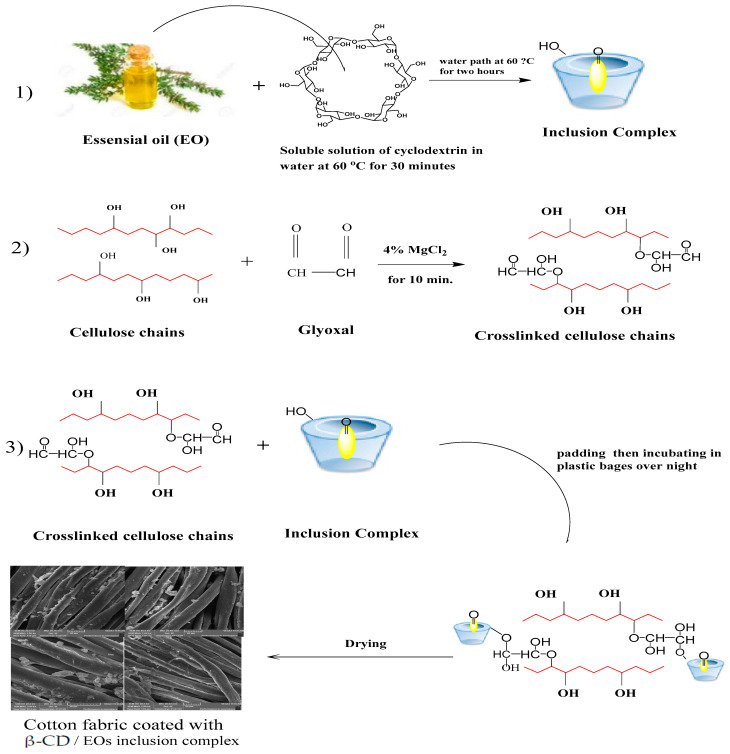
Steps of schematic mechanism for coated cotton fabric with inclusion complexes.

The existence of functional groups on the surface of the fabrics with the inclusion complex β-cyclodextrin (β-CD)/essential oils (β-CD-EO_S_) was identified by the FTIR spectra in the range from 400 to 4000 cm^−1^ (Figure 1). Figure 1 demonstrates the FTIR spectra for the two methods under study compared with that of the blank cotton substrate. According to methods 1 and 2, the spectrum revealed both an increase and a drop in the vibrational intensity of the existing peaks. On the spectral bands of the cotton sample treated with the inclusion complex of β-CD-EO_S_, differences could be observed. It showed that cotton treated with β-CD-EO_S_ inclusion complexes had an additional peak at 1749 cm^−1^ for method 1 and a peak at 1734 cm^−1^ for method 2, which was not apparent in untreated fabric. The presence of a strong carbonyl group C=O confirmed the β-CD-EO_S_ inclusion complexes deposited on the surface of the fabric [27]. A number of shifts in the treated samples’ spectral peaks were noticed in the range of 1451 to 700 cm^−1^ for method 1 and spectral peaks in the range of 1457 to 710 cm^−1^ for method 2, depending on the type of EO_S_ used and conditions of treatments. Furthermore, the peaks at 1451 and 1457 cm^−1^ for methods 1 and 2 could be attributed to the C=C elongation vibrations of essential oil oleophilic groups [28]. As previously mentioned, inclusion complexes are created by interactions between the guest and host molecules. In addition, they are involved in hydrogen bonds, Van der Waals forces, interactions involving hydrophobicity, and the release of high-energy water molecules from CD cavities [29]. The hydrophobic group with less polarity enters the cavity of cyclodextrin, while the hydroxyl group from the essential oil remains outside. The hydroxyl group outside of the cyclodextrin created an intermolecular hydrogen bond with −OH, and this structure can increase the stability of the entire inclusion complex system (Scheme 3, step 1) [30]. The formed inclusion complexes can form a stable chemical bond with the crosslinked cotton fabric via ether linkage formed between the glyoxal and the hydroxyl group of the cotton cellulose (Scheme 3 steps 2 and 3) [31]. It was noticeable that an ether bond disappeared in the FTIR spectra for both methods 1 and 2. By considering the low concentration of glyoxal solution (4%) and the cross-linking mechanism, the induced formation of C-O bonds in the thin interphase between elementary fibers would be negligible compared with the ether functions that are already present in the ß-1,4 D-glucose units of cellulose. The functional groups generated by the reaction simply overlap those in the intrinsic structure of the cotton fibers [32]. The strong intensity of the peaks in method 2 (pad-batch) rather than in method 1 (pad-dry–cure) is also notable from Figure 1. This could have been responsible for the batch process enhancing interaction between the β-CD-EO_S_ inclusion complexes and the cellulose fiber [33].

### 4.2. SEM Analysis

**Figure 2:**
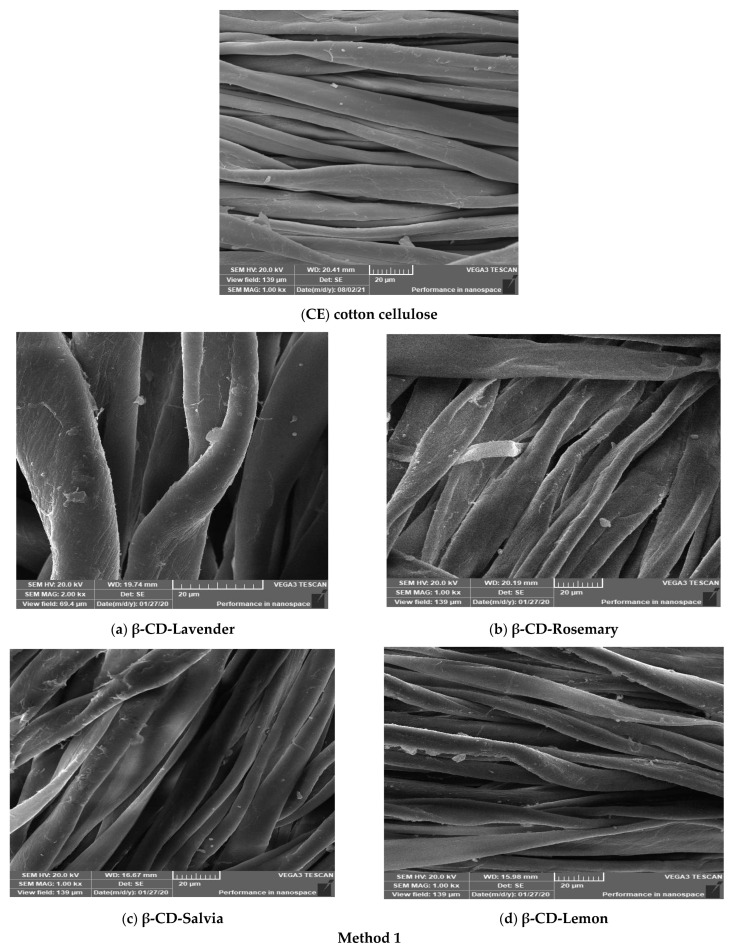

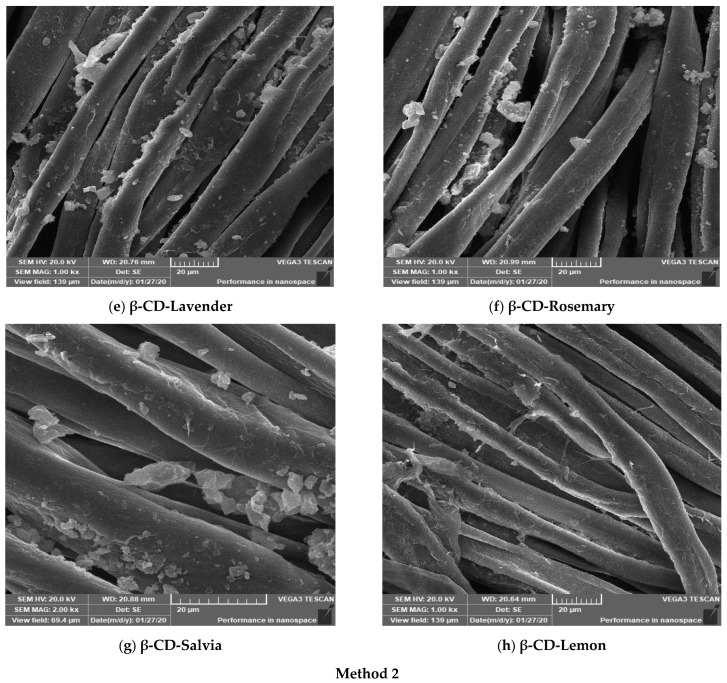
SEM images of blank cotton cellulose and β-CD-EO_S_ treated samples.

Figure 2 depicts the SEM morphology of the blank cotton cellulose (CE) and cotton textiles impregnated with β-CD-EO_S_ inclusion complexes using methods 1 (a, d, c, d) and 2 (e, f, g, h). Figure 2 CE indicates that the surface appearance of the blank cotton fabric was smooth and flat with small twists [34,35]. The surface of the cotton fabric coated with the β-CD-EO_S_ inclusion complexes had small particles scattered over it (a, d, c and d) for method 1. However, the surface of the fabric had a significant number of particles accumulated and aggregated on the surface of the samples (e, f, g, and h) for method 2. The inclusion complexes deposited to the fabric covered its surface, allowing the finished layer to form. This revealed that both methods 1 and 2 were effective for applying inclusion complexes to cotton fabrics.

### 4.3. Antibacterial Activity

The antibacterial activity of the fabrics treated with various β-CD-EO_S_ inclusion complexes were carried out against two types of Gram-positive (*S. aureus* and *B. subtilis*) and two types of Gram-negative (*E. coli* and *P. aeruginosa*) strains. Table 1 illustrates the antibacterial activity for treated fabrics via method 1 and method 2.

The results of Table 1 were summarized and revealed that:

- All cotton fabric treated by either method 1 or method 2 exhibited high antibacterial activities.
- The cotton samples treated by pad batch (method 2) showed higher antibacterial properties. This may have been due to the effect of the high temperature used in pad-dry-cure (method 1) on the encapsulation process, and the rapid release of EOs from the fabric, consequently reducing its bioactivity to some extent. This is in agreement with the results obtained in a previous study [36].
- The antibacterial activities of the treated samples varied according to the nature of EOs. They followed the order: β-CD/Lavender ˃ β-CD/Rosemary~β-CD/Lemon ˃ β-CD/Salvia.
- The bioactive constituents of EO_S_, such as terpenes and their oxygenated derivatives, are responsible for their biological properties.
- The mechanism of their activity could be attributed to their hydrophobic nature, which enables them to interact with the lipids found in the cell membranes of bacteria. This increases susceptibility by disrupting cell structures as they bind to the cell’s surface and subsequently penetrate the cell membrane’s phospholipid bilayer. Their accumulation affects the cell membrane’s structural stability, which can have a severe effect on cell metabolism and cause cell death. This finally leads to the death of the bacteria cell due to the significant leaking of its essential components and ions [37].

**Figure 3:**
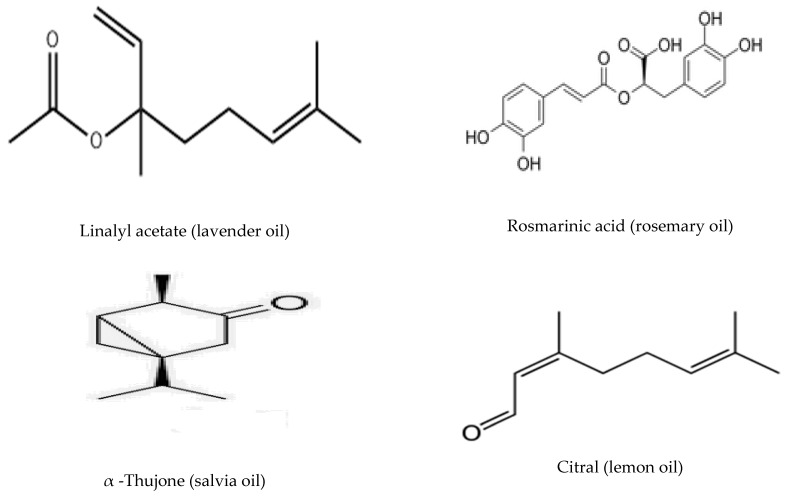
Structures of the main compounds of essential oils.

In addition, the biological and chemical activities of the essential oils depended on the chemical compounds present in the isolated essential oils (Figure 3). The inhibitory effect of rosemary was the result of the action of rosmarinic acid, rosmaridiphenol, carnosol, epirosmanol, carnosic acid, rosmanol, and isorosmanol [11]. High and almost equal contents of linalool and linalyl acetate (a ratio above one) were required for good antimicrobial properties of lavender essential oil [10]. They interacted with the cell membrane, causing changes in genetic material and nutrients, altering the transport of electrons, leakage of cellular components, and production changes in fatty acid. In addition, they produced an interaction with the membrane of proteins that produced the loss of membrane functionality and its structure. Moreover, the antimicrobial activity of the essential oils of C. limon are apparently related to its terpene’s type components, such as pinene, myrcene, and limonene [13]. Also the antibacterial action of salvia essential oils is related to its components, such as cineole, α-thujone and camphor [12]. 

Finally, the inhibitory activity of an essential oil is known to result from a complex interaction between its different constituents, which may produce additive, synergistic, or antagonistic effects, even for those present at low concentrations.

The results also demonstrated that Gram-positive bacteria were more resistant to EOs [38]. The outer membrane of Gram-negative bacteria is rigid, rich in lipopolysaccharide (LPS), and more complex, limiting the diffusion of hydrophobic compounds through it, while the outer membrane of Gram-positive bacteria is surrounded by a peptidoglycan wall that is not dense enough to resist small antimicrobial substances, allowing for easier access to the cell membrane [37]. Furthermore, the lipophilic ends of lipoteichoic acid prevalent in Gram-positive bacteria cell membranes may assist the entry of hydrophobic EO components [39].

Table 1 shows the resistance to washing cycles. Increasing the number of washing cycles to five clearly resulted in a minor decline in the antibacterial activities of the treated samples. This may have been due to the influence of the crosslinkers utilized (glyoxal) [40,41]. As a result, washing durability was improved.

### 4.4. Scent Intensity

For this evaluation, four healthy judges were chosen in accordance with the human subject guidelines of Eastern Michigan University. The judges smelled the samples every 5 days for a total of 30 days. The intensity of the specimen’s smell was measured after each wash [42]. Table 2 shows the scent intensity of cotton fabric treated with β-CD-EO_S_. The rating system utilized was ordinal, with zero denoting no scent and five denoting a very strong aroma [43]. The samples were washed six times at 50 °C with tide commercial detergent, and air-dried at ambient conditions. The scent strength was quite similarly assessed by the judges for all samples. The presence of β-CD influenced the fragrance released from the fabric. In general, cotton fabric provided more rating points for all scents, especially for fabrics treated with method 2. This occurrence was due to a greater weight gain of the β-CD on the cotton fabric, which helped it to absorb extra essential oil aromas. The fabrics were rated for the first time the day after the aromas were applied, and the final rating was assigned after 30 days and six washes. As indicated, a general trend of declining ratings was noticed over time for all the treated fabric with aromas. The rating results demonstrated no significant difference in the intensity of the scents produced by the various types of oils utilized. The scents’ presence substantially reduced after 30 days and six washes, but they did not completely disappear. Generally, cotton fabrics treated by method 2 had higher scent intensity than fabric treated by method 1. The order of scent intensity of oils used was as follows: β-CD/Lavender **˃ β**-CD/Lemon **˃** β-CD/Rosemary **˃** β-CD/Salvia.

### 4.5. Weight Gain, Tensile Strength, and Air Permeability Measurements

The mechanical characteristics of cotton fabric impregnated with β-CD/EOs are shown in Table 3, together with the percentage of values for weight gain measurements. The weight gain reflected the amount of chemicals that were coated on the fabric during treatment. The results showed that the weight gain values for the β-CD/EO_S_-treated fabric by method 1 were between 8.65% and 10.55%, whereas the treatment of fabrics with method 2 caused an increase in the weight gain ranging between 9.01% and 11.58%.

In addition, Table 3 shows slight increases in the tensile strength values of the finished fabrics treated with the pad-dry-cure process (method 1). It was also observed that there was a slight decrease in tensile strength when the fabric samples were treated with the pad-batch method (method 2). The ether bond between the cotton hydroxyl groups and β-CD/EO_S_, which reduced the restriction on the segmental movement of cellulose chains and protected the fiber from being very tender, was responsible for the improvement in strength [44]. Conversely, the slight decrease in strength was due to various changes and crosslinking agents, leading to weakened cellulose chains.

Table 3 illustrates the air permeability of the treated fabric. It is regarded as a significant factor in the treated textiles’ breathability. Along with the data shown in the table, it is evident that the treated fabrics had slightly lower air permeability values. This may have been related to the different treatments of coating covering open interstices [45]. Furthermore, the swelling of hydrophilic cotton could have altered the fabric’s porosity and thickness, causing a reduction in the treated fabrics’ air permeability [46].

## 5. Conclusions

Essential oils are compounds of great importance. In this study, we considered the inclusion complexes between four different essential oils and β-CD, aiming to increase the wash resistance of antibacterial cotton fabric. Two different successful treatments of the cotton fabric were developed for enhancing the inclusion complexes’ bonding.

The results indicate that the pad-batch method (2) was more efficient than the pad-dry-cure method (1). For scent intensity, the order of oils used was as follows: β-CD/Lavender ˃ β-CD/Lemon ˃ β-CD/Rosemary ˃ β-CD/Salvia. Moreover, all treated samples had higher durable antibacterial efficiency for Gram-positive than Gram-negative bacteria, and varied according to the nature of the EOs. It followed the order: β-CD/Lavender ˃ β-CD/Rosemary~β-CD/Lemon ˃ β-CD/Salvia. The mechanical properties of the treated samples were slightly affected. The outcome of this study is an innovation that may be used to develop customized aroma fabrics and provide new insight into both finishing textiles with fragrances and medical research.

## Figures and Tables

**Table 1:** Antibacterial activity and durability properties for different fabrics’ treatments against different bacterial species.

Treatment of Fabric		Inhibition Zone (mm/1 cm Sample)							
		G^+^				G^−^			
		*Bacillus subtilis*		*Staphylococcus aureus*		*Escherichia coli*		*Pseudomonas aeruginosa*	
No. of washing cycles		1	5	1	5	1	5	1	5
Untreated cotton fabric		0	0	0	0	0	0	0	0
Method (1)	β-CD/Lavender	33 ± 0.57	24 ± 0.28	31 ± 0.28	24 ± 0.57	32 ± 0.28	29 ± 0.28	31 ± 0.52	26 ± 0.57
	β-CD/Rosemary	29 ± 0.57	23 ± 0.57	28 ± 0.57	21 ± 0.57	32 ± 0.52	28 ± 0.28	30 ± 0.57	24 ± 0.57
	β-CD/Salvia	26 ± 0.57	20 ± 0.28	25 ± 0.57	19 ± 0.28	30 ± 0.57	24 ± 0.23	28 ± 0.25	22 ± 0.57
	β-CD/Lemon	30 ± 0.28	21 ± 0.75	30 ± 0.28	20 ± 0.5	31 ± 0.28	27 ± 0.2	29 ± 0.25	23 ± 0.25
Method (2)	β-CD/Lavender	39 ± 0.20	30 ± 0.28	34 ± 0.76	26 ± 0.25	35 ± 0.28	30 ± 0.25	33 ± 0.20	29 ± 0.20
	β-CD/Rosemary	36 ± 0.57	28 ± 0.28	32 ± 0.50	24 ± 0.28	33 ± 0.28	30 ± 0.28	30 ± 0.57	25 ± 0.20
	β-CD/Salvia	29 ± 0.25	22 ± 0.57	28 ± 0.28	20 ± 0.17	30 ± 0.28	27 ± 0.51	28 ± 0.52	23 ± 0.51
	β-CD/Lemon	32 ± 0.57	23 ± 0.75	31 ± 0.76	22 ± 0.17	32 ± 0.57	25 ± 0.20	31 ± 0.76	24 ± 0.57

**Table 2:** Scent intensity of treated cotton fabrics with inclusion complex β-CD-EOs via methods 1 and 2 rating by judges.

Method	Evaluation Day	β-CD/Lavender	β-CD/Rosemary	β-CD/Salvia	β-CD/Lemon
**Method** **(1)**	1	4.75	4.50	4.25	3.75
	5	4.50	4.50	4.25	3.50
	10	4.25	4.00	4.00	3.25
	15	4.00	3.75	3.75	3.25
	20	3.50	3.50	3.25	3.00
	25	3.25	3.00	3.00	2.75
	30	3.00	2.25	2.00	2.75
**Method** **(2)**	1	5.00	4.75	5.00	5.00
	5	5.00	4.50	4.75	4.75
	10	4.50	4.25	4.50	4.75
	15	4.50	3.75	3.75	4.25
	20	4.00	3.50	3.25	3.75
	25	3.50	3.00	3.00	3.50
	30	3.25	2.50	2.25	3.00

**Table 3:** Weight gain, tensile strength, and air permeability measurements of cotton fabric treated with inclusion complexes via methods 1 and 2.

Treatment of Fabric		Weight Gain (%)	Tensile Strength (kg f)	Air Permeability (cm^3^/cm^2^/s)
Untreated cotton fabric (CE)		0	56 ± 0.66	28.56
Method (1)	β-CD/Lavender	10.55 ± 0.1	56 ± 0.76	25.24
	β-CD/Rosemary	9.55 ± 0.06	57 ± 1.50	26.22
	β-CD/Salvia	8.65 ± 0.05	58 ± 0.36	23.23
	β-CD/Lemon	9.65 ± 0.4	56 ± 0.55	27.48
Method (2)	β-CD/Lavender	11.58 ± 0.5	55 ± 0.05	26.76
	β-CD/Rosemary	10.02 ± 0.04	55 ± 0.21	26.18
	β-CD/Salvia	9.01 ± 0.02	54 ± 0.87	24.21
	β-CD/Lemon	9.32 ± 0.23	53 ± 0.68	27.18

## Data Availability

The data presented in this study are available on request from the corresponding author.

## References

[1] Guerra-Rosas M., Morales-Castro J., Cubero-Márquez M., Salvia-Trujillo L., Martín-Belloso O. (2017). Antimicrobial activity of nanoemulsions containing essential oils and high methoxyl pectin during long-term storage. Food Control.

[2] Abd El-Hady M.M., Farouk A., Saeed S.E.S., Zaghloul S. (2021). Antibacterial and UV Protection Properties of Modified Cotton Fabric Using a Curcumin/TiO2 Nanocomposite for Medical Textile Applications. Polymers.

[3] Islam S., Shahid M., Mohammad F. (2013). Perspectives for natural product based agents derived from industrial plants in textile applications—A review. J. Clean. Prod..

[4] Solorzano-Santos F., Miranda-Novales M.G. (2012). Essential oils from aromatic herbs as antimicrobial agents. Curr. Opin. Biotechnol..

[5] Kavetsou E., Pitterou I., Katopodi A., Petridou G., Adjali A., Grigorakis S., Detsi A. (2021). Preparation, Characterization, and Acetylcholinesterase Inhibitory Ability of the Inclusion Complex of β-Cyclodextrin–Cedar (*Juniperus phoenicea*) Essential Oil. Micro.

[6] Sell C.S. (2006). The Chemistry of Fragrances: From Perfumer to Consumer.

[7] Pophof B., Gert S., Leif A. (2005). Volatile organic compounds as signals in a plant–herbivore system: Electrophysiological responses in olfactory sensilla of the moth Cactoblastis cactorum. Chem. Senses.

[8] Dhifi W., Bellili S., Jazi S., Bahloul N., Mnif W. (2016). Essential Oils’ Chemical Characterization and Investigation of Some Biological Activities: A Critical Review. Medicines.

[9] Mehalaine S., Belfadel O. (2018). Chemical composition and antibacterial activity of essential oils of three medicinal plants from Algerian semi-arid climatic zone. Phytothérapie.

[10] Białoń M., Krzyśko-Łupicka T., Nowakowska-Bogdan E., Wieczorek P.P. (2019). Chemical composition of two different lavender essential oils and their effect on facial skin microbiota. Molecules.

[11] Nieto G., Ros G., Castillo J. (2018). Antioxidant and antimicrobial properties of rosemary (*Rosmarinus officinalis*, L.): A review. Medicines.

[12] Khedher M.R.B., Khedher S.B. (2017). Chemical composition and biological activities of Salvia officinalis essential oil from Tunisia. EXCLI J..

[13] Ben Hsouna A., Ben Halima N., Smaoui S., Hamdi N. (2017). Citrus lemon essential oil: Chemical composition, antioxidant and antimicrobial activities with its preservative effect against Listeria monocytogenes inoculated in minced beef meat. Lipids Health Dis..

[14] Cevallos P.A.P., Buera M.P., Elizalde B.E. (2010). Encapsulation of cinnamon and thyme essential oils components (cinnamaldehyde and thymol) in β-cyclodextrin: Effect of interactions with water on complex stability. J. Food Eng..

[15] Stan M., Chirila L., Popescu A., Radulescu D.M., Radulescu D.E., Dinischiotu A. (2019). Essential Oil Microcapsules Immobilized on Textiles and Certain Induced Effects. Materials.

[16] Ribeiro A.C.F., Valente A.J.M., Lobo V.M.M. (2008). Transport properties of cyclodextrins: Intermolecular diffusion coefficients. J. Balk. Tribol. Assoc..

[17] Sharaf S., El-Naggar M.E. (2019). Wound dressing properties of cationized cotton fabric treated with carrageenan/cyclodextrin hydrogel loaded with honey bee propolis extract. Int. J. Biol. Macromol..

[18] Shahid M., Mohammad F. (2013). Green Chemistry Approaches to Develop Antimicrobial Textiles Based on Sustainable Biopolymers, A Review. Ind. Eng. Chem. Res..

[19] Grgac S.F., Malinar R. (2018). Application of Environment-Friendly Processes Aimed at Bonding β-Cyclodextrine onto Cellulosic Materials. Fibres Text. East. Eur..

[20] Agrawal P.B., Warmoeskerken M.M.C.G. A β-cyclodextrin based slow and controlled release system for digital finishing of cotton. Proceedings of the Autex Textile World Conference 2008.

[21] Kfoury M., Landy D., Fourmentin S. (2018). Characterization of cyclodextrin/volatile inclusion complexes: A review. Molecules.

[22] Hadžiabdíc J., Elezovíc A., Rahíc O., Mujezin I. (2012). Effect of Cyclodextrin Complexation on the Aqueous Solubility of Diazepam and Nitrazepam: Phase- Solubility Analysis, Thermodynamic Properties. Am. J. Anal. Chem..

[23] Lis M.J., Carmona Ó.G., Carmona C.G., Bezerra F.M. (2018). Inclusion complexes of citronella oil with β-cyclodextrin for controlled release in biofunctional textiles. Polymers.

[24] Bezerra F.M., Lis M.J., Firmino H.B., da Silva J.G.D., Valle R.d.S.C., Valle J.A.B., Scacchetti F.A.P., Tessaro A.L. (2020). The Role of β-Cyclodextrin in the Textile Industry-Review. Molecules.

[25] Haji A., Shabbir M., Ahmed S., Sheikh J.N. (2020). Functional Finishing of Textiles with β-Cyclodextrin. Frontiers of Textile Materials: Polymers, Nanomaterials, Enzymes, and Advanced Modification Techniques.

[26] Grgac S.F., Jablan J., Inić S., Malinar R., Kovaček I., Čorak I. (2022). The Effect of Ultrasonic Treatment on the Binding of the Inclusion Complex β-Cyclodextrin-peppermint Oil with Cellulose Material. Materials.

[27] Khanna S., Sharma S., Chakraborty J.N. (2015). Performance assessment of fragrance finished cotton with cyclodextrin assisted anchoring hosts. Fash. Text..

[28] Sricharussin W., Sopajaree C., Maneerung T., Sangsuriya N. (2009). Modification of cotton fabrics with β-cyclodextrin derivative for aroma finishing. J. Text. Inst..

[29] Torres-Alvarez C., Castillo S., Sánchez-García E., Aguilera González C., Galindo-Rodríguez S.A., Gabaldón-Hernández J.A., Báez-González J.G. (2020). Inclusion complexes of concentrated orange oils and β-cyclodextrin: Physicochemical and biological characterizations. Molecules.

[30] Zhang T., Luo Y., Wang M., Chen F., Liu J., Meng K., Zhao H. (2020). Double-Layered Microcapsules Significantly Improve the Long-Term Effectiveness of Essential Oil. Polymers.

[31] Hebeish A., Sharaf S., Refaie R., El Shafei A. (2012). Multifinishing of Cotton Fabrics Using Microwave Techniques. Res. J. Text. Appar..

[32] Perremans D., Hendrickx K., Verpoest I., Van Vuure A.W. (2018). Effect of chemical treatments on the mechanical properties of technical flax fibres with emphasis on stiffness improvement. Compos. Sci. Technol..

[33] Shu D., Fang K., Liu X., Cai Y., Zhang X., Zhang J. (2018). Cleaner coloration of cotton fabric with reactive dyes using a pad-batch-steam dyeing process. J. Clean. Prod..

[34] Abd El-Hady M.M., Farouk A., Sharaf S. (2022). Multiwalled-Carbon-Nanotubes (MWCNTs)–GPTMS/Tannic-Acid-Nanocomposite-Coated Cotton Fabric for Sustainable Antibacterial Properties and Electrical Conductivity. Coatings.

[35] El-Sayed Saeed S., Al-Harbi T.M., Abdel-Mottaleb M.S.A., Al-Hakimi A.N., Albadria A.E.A.E., Abd El-Hady M.M. (2022). Novel Schiff base transition metal complexes for imparting UV protecting and antibacterial cellulose fabric: Experimental and computational investigations. Appl. Organomet. Chem..

[36] Suthaphot N., Chonsakorn S., Mongkholrattanasit R. Application of Aromatherapy on Cotton Fabric by Microcapsules. Proceedings of the RMUTP International Conference: Textile and Fashion 2012.

[37] Sharaf S., El-Naggar M.E. (2018). Eco-friendly technology for preparation, characterization and promotion of honey bee propolis extract loaded cellulose acetate nanofibers in medical domains. Cellulose.

[38] Azhdarzadeh F., Hojjati M. (2016). Chemical composition and antimicrobial activity of leaf, ripe and unripe peel of bitter orange (Citrus aurantium) essential oils. Nutr. Food Sci. Res..

[39] Zinoviadou K.G., Koutsoumanis K.P., Biliaderis C.G. (2009). Physico-chemical properties of whey protein isolate films containing oregano oil and their antimicrobial action against spoilage flora of fresh beef. Meat Sci..

[40] Panigrahi D., Kumar S., Dhar A. (2019). Modulating chain conformations of polyvinyl alcohol through low cost and nontoxic glyoxal crosslinker: Application in high performance organic transistors. Org. Electron..

[41] Sharaf S., Higazy A., Hebeish A. (2013). Propolis induced antibacterial activity and other technical properties of cotton textiles. Int. J. Biol. Macromol..

[42] Ghosh S., Chipot N. (2015). Embedding aromatherapy essential oils into textile fabric using β-cyclodextrin inclusion compound. Indian J. Fibre Text. Res..

[43] Muresan A., Cerempei A., Dunca S., Muresan R., Butnaru R. (2009). Aromatherapeutic characteristics of cotton fabrics treated with rosemary essential oil. Cellul. Chem. Technol..

[44] Wang H., Wei D., Ziaee Z., Xiao H., Zheng A., Zhao Y. (2015). Preparation and properties of nonleaching antimicrobial linear low-density polyethylene films. Ind. Eng. Chem. Res..

[45] Farouk A., Moussa S., Ulbricht M., Textor T. (2012). ZnO Nanoparticles-Chitosan Composite as Antibacterial Finish for Textiles. Int. J. Carbohydr. Chem..

[46] Afzal A., Hussain T., Malik M.H., Rasheed A., Ahmad S., Basit A., Nazir A. (2014). Investigation and modeling of air permeability of Cotton/Polyester blended double layer interlock knitted fabrics. Fibers Polym..

